# Telephonic nursing intervention for laparoscopic cholecystectomy and hernia repair: A randomized controlled study

**DOI:** 10.1186/s12912-020-00432-y

**Published:** 2020-05-11

**Authors:** Renata da Silva Schulz, Rosimere Ferreira Santana, Carla Targino Bruno dos Santos, Thiago Batista Faleiro, Dayana Medeiros do Amaral Passarelles, Ana Beatriz Serra Hercules, Thalita Gomes do Carmo

**Affiliations:** 1 Jorge Amado University, Salvador, BA Brazil; 2 Federal Fluminense University, CNPq researcher, Rio de Janeiro, Brazil; 3 Present Address: Rua Dr. Celestino, 74, 6° andar, Niterói, Rio de Janeiro, CEP: 24020-091 Brazil; 4 University of Brasilia, Federal District, Brasilia, Brazil; 5 Clinical Hospital of Salvador, Brasilia, BA Brazil; 6 Fluminense Federal University, Rio de Janeiro, Brazil

**Keywords:** Home telecare, Telegeriatrics, Telenursing, Teleconsulting, Telesurgery, Geriatric nursing

## Abstract

**Background:**

Patient undergoing surgery may be afraid and concerned about the diagnosis, the treatment, the procedure, the postoperative care, and the surgical recovery. Good communication between staff and patients can minimize or prevent this situation. This study aimed to evaluate the effectiveness of a Telecare nursing intervention, “Telephone consultation”, in reducing the “Delayed surgical recovery” nursing diagnosis in patients undergoing laparoscopic cholecystectomy and hernia repair.

**Methods:**

This study was performed in two different institutions located in Rio de Janeiro, Brazil. A total of 43 patients were enrolled. The experimental group consisted of 22 patients who had access to the telephone follow-up intervention, and the control group consisted of 21 patients who received conventional treatment without telephone follow-up. This was a randomized controlled study with patients who were 60 years or older and awaiting operative procedures of hernia repair and laparoscopic cholecystectomy who had a mobile or landline phone and were available for telephone contact.

**Results:**

There was a reduction in “loss of appetite with nausea” (*p* = 0.013); “need help to complete self-care” (*p* = 0.041); “pain” (*p* = 0.041); and “postoperative sensation” (*p* = 0.023). The experimental group showed a significantly larger decrease in factors related to the “Delayed surgical recovery” diagnosis, suggesting a positive effect of the intervention compared to the effect in control group.\.

**Conclusion:**

Telephone consultation identified factors that increased the risk of complications after surgery, recognized potential patients for delayed surgical recovery and helped perioperative nurses provide accurate interventions to prevent or mitigate delayed recovery.

This study was registered in the platform Brazilian Registry of Clinical Trials (ReBEC) - link: http://www.ensaiosclinicos.gov.br under registration number RBR-4C249M, retrospectively registered on April 13, 2020.

## Background

The occurrence rate of perioperative adverse events is between 3 and 16% in surgical procedures, and nearly 7 million patients suffer significant complications each year [1]. Half of these complications can be avoided by improving communication and thereby reducing injuries in patients [1, 2]. The surgical procedure of operating frightens patients who might be afraid of the surgery itself or are concerned about the diagnosis, the treatment, the procedure, the postoperative care, and other factors [3]. These factors can be minimized or avoided by good communication practices between staff and patients.

Data show that 74% of patients preferred guidance on how to take care of themselves at home. However, only 57% said they received counseling at discharge. Among them, 52.2% received medical advice, and 43.5% did not remember receiving guidelines [4]. It is important to reduce the postoperative hospitalization time to prevent infection of surgical wounds, prevent complications associated with prolonged immobilization and to minimize healthcare costs. Therefore, it is necessary to create guidelines for home care and promote a better relationship between patients and institutions, which is a challenge [5]. The incomplete understanding of the care instructions may affect the patient’s recovery. Monitoring these patients to efficiently detect problems at an early stage can be a strategy for postoperative follow-up [6].

“Telephone Consultation” (8180) is a nursing intervention established by the Nursing Interventions Classifications (NIC) with the goals of monitoring the health conditions of a patient and taking action in abnormal situations. This type of intervention is conducted in collaboration with face-to-face nursing consultation and is complementary to postoperative care but does not replace it. Moreover, telephone consultation can reduce the anxiety of patients and take away any doubts in a limited amount of time, resulting in an increased intensity of the bond with professionals and the satisfaction of patients who receive care. “Telephone Consultation” was primarily designed for the follow-up of patients with chronic diseases, but a few studies report use of this intervention for surgical conditions such as urologic procedures, breast reconstruction, hip surgery, and heart surgery [7–11].

For this study, we used the diagnosis “Delayed Surgical Recovery” (NANDA-I: 00100). This diagnosis is part of the “Safety and Protection, Physical Injury” category and is defined as an “extension of the number of postoperative days required to initiate and perform activities that maintain life, health, and well-being” [12]. The following items are described as factors related to this diagnosis: an extensive surgical procedure, obesity, pain, preoperative expectations, postoperative surgical site infection, and a prolonged surgical procedure [12]. The defining characteristics of this diagnosis include the following: difficulty moving around, evidence of interrupted healing of the surgical area, fatigue, loss of appetite with or without nausea, a perception that more time is needed to recover, postponed resumption of work/employment activities, reported pain or discomfort, and requiring help to complete self-care [12].

The prevalence rate of “Delayed Surgical Recovery” in a previous study was 37% among adults and the elderly, and of all types of surgery, patients with gastrointestinal surgery had the highest prevalence (31%) [13]. This study aimed to evaluate the effectiveness of a Telecare nursing intervention, “Telephone consultation”, in reducing the “Delayed surgical recovery” nursing diagnosis in patients who are undergoing laparoscopic cholecystectomy and hernia repair.

## Methods

This study was conducted at Antônio Pedro University Hospital and at the Sevidores State Hospital, both located in Rio de Janeiro/Brazil, during the period of March to August 2016. The follow-up time for each participant was 4 weeks because the laparoscopic cholecystectomy and hernia repair were considered acute conditions with a recovery time of approximately 7 days. Patients who still experienced pain, were not able to walk on their own, who needed help to complete everyday life tasks and who did not fully recover from surgery within 1 week were considered to have delayed surgical recovery, which was the main outcome of the study [14]. Two follow-ups occurred in parallel: DSR diagnosis identification follow-up of all participants and telephone follow-up of the experimental group.

The inclusion criteria were as follows: people aged 60 years or older; patients preoperative for laparoscopic cholecystectomy and hernia repair; possession of a telephone or cellphone; and patients available for the nurse’s calls. The exclusion criteria were patients diagnosed with dementia, patients with hearing problems without caregivers who could receive interventions over the phone, and patients undergoing surgery for the treatment of surgical complications. The study discontinuity criteria were as follows: not answering 75% of the nurse’s calls or not having time to receive the interventions over the phone.

The follow-up for the DSR diagnosis was conducted by 4 examiners who were trained to determine the diagnosis of “Delayed Surgical Recovery”. The training had three phases: the first phase was about presenting the research protocol, the second phase presented the evaluation of each variable, and the third phase determined the accuracy of all variables used in this research in a pilot study of twelve study cases. The variable assessment instrument, which is described below, was applied in both the experimental and control groups. At the end of the follow-up, an educational primer on postoperative care for the elderly developed by the research team was distributed to both to the experimental group and the control at the time of hospitalization to make the groups as homogeneous as possible.

The research instrument was constructed to ensure that the data collection was performed in a standardized way according to the conceptual and operational definitions of each clinical indicator. These definitions were developed based on other institutional protocols available in articles, books and manuals as described below [15].

1. Discomfort: Determined from the physical examination and verbal report of the patient.
2. Evidence of interrupted healing at the surgical site: Determined from physical examination or the medical records.
3. Surgical site infection: Determined by the MagedanzSCORE.
4. Loss of appetite: Determined from the Mini Nutritional Assessment (MNA) test.
5. Need for assistance with self-care: Determined from the Barthel Index.
6. Impaired mobility: Determined from the Barthel Index.
7. Edema at the surgical site: Determined by the Godet sign.
8. Diabetes mellitus: Determined from the medical records.
9. Persistent nausea: Simplified Apfel scale.
10. Persistent vomiting: Simplified Apfel scale.
11. Old age: Age ≥ 85 years based on medical records.
12. History of delayed wound healing: Reports of “operative wound dehiscence” in a previous surgery.
13. Pain: Visual analog scale.
14. Malnutrition: Serum albumin ≤3.8 and body mass index< 18.5.
15. Obesity: Body mass index ≥30 and < 34.9 kg/m2.
16. Postoperative emotional response: Medical records.
17. Trauma at the surgical site: Physical examination and skin inspection.

Telephone follow-up was performed by the researcher who knew the sample group assignments. The instrument for this monitoring was elaborated through questions and guidelines for home care. The instrument included a final moment to withdraw doubts from the elderly regarding the surgical process.

A simple randomization of the sample was performed using the Statistical Package for Social Sciences (SPSS) used for the randomized group allocation; 22 participants were assigned to the experimental group and 21 participants were assigned to the control group. The principal investigator applied the randomized allocation sequence to the study participants to carry out the intervention and kept the randomization results in her exclusive possession during the research.

The data collection was conducted from March to August 2015. The study was performed at 2 university hospitals located in the state of Rio de Janeiro. A total of 43 patients were enrolled.

The experimental group received the “Telephone Consultation” intervention from a researcher on the 4th (D4), 8th (D8), 12th (D12), 18th (D18) and 25th (D25) postoperative day; a total of 5 telephone consultations were attempted for each participant in the experimental group. During the patient’s follow-up, we used the guidelines developed by NIC standardization and a literature review (e.g., questions about mobility at home, food intake and wound care). The control group was evaluated during the hospitalization time (D2) and at regular consultations (D15, D30). The experimental group was also evaluated at these time points.

The statistical analysis included the chi-square test (χ^2^) or Fisher’s exact test for categorical data and Student’s t test for independent samples. To analyze the evolution of the defining characteristics and related factors of the DSR diagnosis, the McNemar corrected test was applied. All statistical analyses were processed using the SAS® System (version 6.11) statistical software.

## Results

### Participant flow

**Fig. 1:**
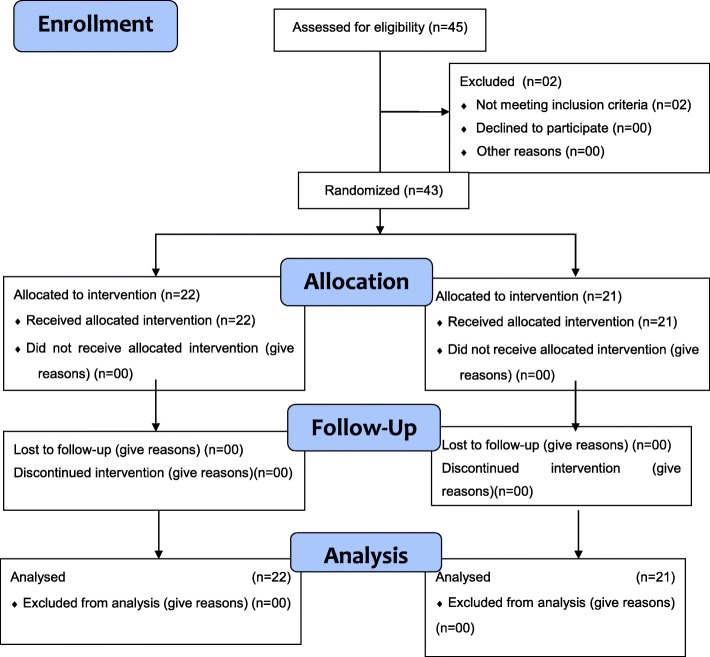
Flow diagram of participants

The total number of participants approached to carry out this study was 45. Of this total, two participants who did not meet the inclusion criteria were excluded: one, because they were undergoing a repeat surgical approach and the other did not have a phone to answer the calls. Thus, a sample of 43 participants, 22 in the experimental group and 21 in the control group, were randomized and followed. There was no loss to follow-up in either group. Figure 1 presents the CONSORT flow diagram of the participants throughout the study.

### Recruitment

The recruitment period was March–August 2015. The participants were followed for 4 weeks. The conventional follow-up in the experimental group and control group occurred on the preoperative day, on the 2nd, 15th and 30th postoperative days, while the experimental group also received follow-up calls on the 4th, 8th, 12th, 18th and 25th postoperative days. There was no loss of follow-up in either group.

### Baseline data

To verify whether the sample was homogeneous, the characteristic profile of the participants was evaluated. The control and experimental groups had the same distribution of characteristics, i.e., the same baseline initial conditions. Table 1 provides information regarding the characterization of the sample.

**Table 1:** Social and Demographic Variables per Group

Variable	Category	Group A (*n* = 22)		Group B (*n* = 21)		*p* value^a^
		N	%	N	%	
Sex	Male	9	40.9	9	42.9	0.900
	Female	13	59.1	12	57.1	
Marital Status	Single	4	18.2	6	28.6	0.720
	Married	12	54.6	10	47.6	
	Widow	5	22.7	3	14.3	
	Divorced	1	4.6	2	9.5	
Education	Illiterate	3	13.6	1	4.8	0.550
	Incomplete elementary school	12	54.6	9	42.9	
	Complete elementary school	1	4.6	2	9.5	
	Incomplete high school	5	22.7	5	23.8	
	Complete high school	1	4.6	4	19.1	
Income	Retired	13	59.1	11	52.4	0.480
	Pensioner	5	22.7	2	9.5	
	Homemaker	2	9.1	4	19.1	
	Working	2	9.1	4	19.1	
Comorbidities	HTN	12	60.0	8	47.1	0.610
	DM	4	20.0	3	17.7	
	HTN + DM	4	20.0	6	35.3	
Obesity	A	25.6 ± 3.7 (19.4–32.9) * kg/m2				
	B	25.9 ± 3.3 (20.3–32.5) * kg/m2				
Age (years)		69.2 ± 7.4 (60–86)*		69.5 ± 8.4 (60–90)*		

*expressed as the average and standard deviation (minimum - maximum); *p* value^a^ ***=*** Fisher exact test; *A* Experimental group; *B* Control group; *n* Frequency; *%* Percentage; *HTN* Hypertension and *DM* Diabetes mellitus

In both groups, the most frequent social and demographic variable categories were married, incomplete elementary school, income from retirement and hypertension. There was no significant difference in the sociodemographic variables between the experimental and control groups at baseline.

### Comparison of clinical indicators associated with the delayed surgical recovery diagnosis

Table 2 compares the defining characteristics related to the diagnosis of “Delayed Surgical Recovery” between the groups. There was a significant decrease from the 1st to the 2nd evaluation (*p* = 0.077) and from the 1st to the 3rd evaluation (*p* = 0.013) for the defining characteristic “loss of appetite with nausea” in the experimental group. For the other defining characteristic, “need help to complete self-care,” there was a significant decrease in the control group (*p* = 0.041) from the 1st to the 3rd evaluation.

**Table 2:** Comparison of the Defining Characteristics for “Delayed Surgical Recovery” Between the Groups

Defining characteristics	Group	(D2)		(D15)		(D30)	*p* value^a^	
		%	%	N	%	D2 x D15	D2 x D30	D15 x D30
Postponed return to work/employment activities	A	–	18.2	1	4.6	*	*	0.240
	B	–	23.8	2	9.5	*	*	0.240
Difficult to move	A	3.6	4.6	1	4.6	0.470	0.470	NP
	B	9.1	14.3	1	4.8	1.000	0.240	0.470
Fatigue	A	9.1	0	0	0	0.470	0.470	NP
	B	19.1	4.8	0	0	0.370	0.300	1.000
Perception that more time is required for recovery	A	9.1	4.6	0	0	1.000	0.470	1.000
	B	14.3	28.6	1	4.8	0.440	0.610	0.130
Evidence of interrupted healing of surgical area	A	0	9.1	0	0	0.470	NP	0.470
	B	0	19.1	1	4.8	0.130	1.000	0.240
Loss of appetite with nausea	A	36.4	9.1	0	0	0.077	0.013	0.470
	B	19.1	14.3	0	0	1.000	0.130	0.240
Loss of appetite without nausea	A	9.1	0	0	0	0.470	0.470	NP
	B	14.3	0	0	0	0.240	0.240	NP
Help needed to complete self-care	A	22.7	9.1	0	0	0.240	0.073	0.470
	B	28.6	14.3	0	0	0.370	0.040	0.240

*p* value^a^ = corrected McNemar test; groups: *A* Experimental group and *B* Control group; *%* Percentage; *NP* Not performed; *D2* 2nd Day after surgery; *D15* 15th Day after surgery and *D30* 30th Day after surgery

Overall, there were differences between the 2 groups regarding the defining characteristics on the 15th day (*p* = 0.03). The percentage of patients with a “perception that more time is needed to recover” was reduced in the experimental group compared to the control group (*p* = 0.046).

Table 3 provides the frequency (n) and percentage (%) of related factors, that is, causal factors related to the Delayed Surgical Recovery diagnosis, at the 3 evaluations. A significant reduction from the 1st to the 3rd evaluation visit was observed in both groups for the “pain” variable (*p* = 0.041) and was observed in the experimental group for the “postoperative expectations” (*p* = 0.023) variable. Patients were positive for the related factor “postoperative expectations” when they presented with anxiety, worry, fear, fear of death, social rejection, the body’s own rejection, insecurity, guilt or loss, mutilated body and decreased self-esteem.

**Table 3:** Comparison of the related factors for “Delayed Surgical Recovery” between groups

Related factor	Group	(D2)		(D15)		(D30)		*p* value^a^	
		%	N	%	N	%	D2 x D15	D2 x D30	D15 x D30
Pain	A	31.8	2	9.1	1	4.6	0.130	0.041	1.000
	B	23.8	3	14.3	2	9.5	0.720	0.440	1.000
Postoperative expectations	A	31.8	3	13.6	0	0	0.280	0.023	0.240
	B	19.1	5	23.8	2	9.5	1.000	0.680	0.370
Postoperative infection at the incision site	A	0	2	9.1	0	0	0.470	NP	0.470
	B	0	4	19.1	1	4.8	0.130	1.000	0.240

*p* value^a^ = corrected McNemar test; groups: A = experimental group and B = control group; *n* Frequency; *%* Percentage; *D2* 2nd Day after surgery; *D15* 15th Day after surgery and *D30* 30th Day after surgery; *NP* Not performed

The related factors “extensive surgery” and “prolonged surgical procedure” were not a part of the analysis in this study because the included surgeries were classified as minor surgeries.

### Cost of the phone calls

In the experimental group, the average total duration of the 5 calls was 37.5 min, and the total call duration ranged from 26 to 53 min. From the 1st to the 5th phone call, there was a reduction in the average call duration from 11.5 to 5.9 min. The total duration of all calls was 826 min, and the average cost of a call to a mobile phone was R$: 0.44, making the total cost R$ 363.44 (approximately USD $114.8) at the time of study analysis.

## Discussion

The experimental group showed a significantly larger decrease in factors related to DSR, suggesting a positive effect of the intervention compared to that in the control group, which was a satisfying study result, considering the sample size. In conclusion, the intervention was feasible and resulted in clinically significant findings. There was no significant difference in social or demographic variables between the 2 groups (*p* > 0.05). In both groups, there was a high comorbidity index, with common chronic conditions such as hypertension and type 2 diabetes mellitus (DM II). It was estimated that 60% of this elderly population had hypertension, and a similar percentage was reported in a study associating the rate of hypertension with diabetes mellitus [16].

The type of surgical procedures was dependent on the patient’s sex: inguinal hernias were more common in men with a proportion of 9:1, while femoral hernias were more common in women with a proportion of 4:1. In this study, surgery was performed for the treatment of inguinal umbilical hernias, which explains the high prevalence of men. Laparoscopic surgery was most prevalent in women [17].

Obesity was a predominant related factor in both groups, and the number of obese women was higher than that of men; obese women constituted 9.3% of the study population, while obese men constituted 4.7%. Obesity can influence the healing process, since excess fat tissue impairs vascularity [18].

The level of education was not significantly different between the groups, but the control group had a slightly higher level of education. According to the interpretation and compliance guidelines, the effectiveness of telecare may be dependent on the level of education. Other studies have described that the higher the patient’s education, the easier it is to understand their pathology, signs and symptoms and the easier it is for them when making decisions to promote, recover and protect their health [19]. In addition, this study demonstrated the phone handling skills of these elderly patients, as only 2 of them had family members take directions.

This study has important implications for perioperative nurses since it helps surgical nurses understand the most relevant components for the prolongation of hospitalization and directs effective interventions during the postoperative period.

The characteristic “needs help to complete self-care” was significantly improved in the control group (*p* = 0.041) over time because most elderly patients needed help with dressing directly after surgery. Another finding was that the defining characteristic “difficult to move” was related to the difficulty in mobilization before the surgery. It is important for nurses to encourage patients and their families to maintain their independence and autonomy. Due to the overall reduced mobility before surgery, only a few patients were mobile by using the lower extremities and ambulating early in the postoperative period [20]. The residences of some patients were unsuitable for performing their activities. Buildings without an elevator and houses on a hillside limited the autonomy of the patient in moving around their home and returning to their social activities.

Another predominant characteristic was “fatigue” evaluated by “prolonged periods of bed rest”, “excessive expenditure of energy while walking” and “fatigue reporting efforts.” There was improvement from the levels of these characteristics before surgery in both groups at the 3rd evaluation visit (D30).

The characteristic “perception that more time for recovery is necessary” is subjective and has been interpreted as the patient feeling weak and insecure about returning to their activities. Although the *p* value did not show significance, the percentage of patients who reported this characteristic decreased from the 1st and the 2nd evaluation visit. This observed difference between the 2 groups is probably due to the health education given on the telephone calls, which resulted in a better understanding of the postoperative process and, consequently, less anxiety and greater security during their postoperative recovery.

A prominent characteristic of “Delayed Surgical Recovery” is “evidence of interruption in surgical healing.” The surgeries were performed according to plan, and thus, in the 1st evaluation visit, no signs of complications were identified. In the 2nd evaluation visit, there were 2 elderly patients in the experimental group and 4 in the control group who had signs of wound exudate, delay in removal of the stitches, and initiation of antibiotic therapy by the assistant surgeon. In the 3rd evaluation visit, only 1 elderly patient in the control group, who underwent treatment for hernia, developed a refractory infectious condition with wound dehiscence.

For the characteristics “loss of appetite with nausea” and “loss of appetite without nausea”, there was a high prevalence in the 1st evaluation visit, which was significantly different in the experimental group (*p* = 0.013); this was mainly caused by the clarification of the use of medication and food. In the experimental group, more information was given on the use of effective antiemetic drugs and the control of environmental factors that can evoke nausea, and the patient was encouraged to breathe deeply and cough, along with other actions. The second, less common reason was the presence of sensory changes and decreased appetite in the elderly, often seen as dissatisfaction with hospital food, which was solved by the return to home, and this reason was absent in the 2nd evaluation visit.

There was a difference in reporting pain in the postoperative period by sex, and women reported experiencing more pain than men. There was a significant decrease in pain in the experimental group (*p* = 0.041). During the intervention, it was noticed that patients did not understand how to correctly take their medications, and after receiving appropriate instructions, many of the patients started to take their prescription properly. Instructions about pharmacological methods for pain relief and education on how to monitor the intensity, quality and duration of pain likely explain the significant improvement in pain relief in the experimental group [21].

Another factor that changed significantly in the experimental group was “postoperative expectations” and complaints about anxiety, worry, and fear. When comparing the percentages, no data on this feature were reported for the last evaluation visit. The *p* value of 0.023 indicated significant findings, whereas in the control group, the *p* value was 0.68. Another related factor contributing to the DSR diagnosis was “postoperative surgical site infection,” and therefore, a major aim of the telephone consultations was to review wound care and observe wound healing [22].

Overall, the factors most important for telecare were wound care, pain control and medication use. However, care related to social needs, such as returning to social activities and decreased anxiety, were also important. Due to technological advances and the influence of improving communication and maintaining social relationships, it has been observed that the use of technology is somewhat ubiquitous among the elderly [23, 24].

Therefore, one generalization of this study is the possibility of telephone consultation; telecare in elderly postoperative patients helped the perception of continuity and supported home care. Among the guidelines made during the intervention, we highlight the promotion of safety and well-being by the follow-up in the postoperative period, offering a sense of continuity of the care provided in the hospital environment and contributing to the patient’s return to daily activities in an expected time.

Regarding study limitations, we point out that this study was not of the multicenter type; it had a considerably small sample with surgeries classified as clean or potentially contaminated. However, this is what guaranteed homogeneity of the sample and the specificity of the intervention. We suggest the continuity of the study in other patient populations and types of surgeries to enhance the intervention in the clinical practice of nursing.

This study’s new knowledge may influence practice:

- Telephone consultations identified factors that increase the risk of complications after surgery.
- Telephone consultations enabled recognition of potential patients for delayed surgical recovery.
- Telephone consultations helped perioperative nurses provide accurate interventions to prevent or mitigate delayed recovery.

## Conclusions

This study showed significantly lower rates for the “loss of appetite with nausea” and “needs help to complete self-care” characteristics and the related factors “postoperative expectations” and “pain” in the intervention group compared to the control group. During the telephone consultations, the main questions involved the use of medications and appropriate wound care.

In conclusion, telephone consultations by nurses are a feasible and helpful intervention for this population in clinical practice due to low cost, positive results, and ease of use. Telephone consultation follow-up is a low-cost resource, accessible to most of the population, with high rates of adherence; it may be used for health care in public health systems worldwide.

## Abbreviations

NIC: Nursing interventions classifications
NANDA-I: North American nursing diagnosis association
DSR: Delayed surgical recovery
SPSS: Statistical package for social sciences
CONSORT: Consolidated standards of reporting trials
DM II: Type 2 diabetes mellitus
